# Pediatric Pleomorphic Xanthoastrocytoma: A National Database Inquiry on Current Treatment Approaches in the United States

**DOI:** 10.1002/cnr2.1415

**Published:** 2021-05-08

**Authors:** Daphne B. Scarpelli, Yun Yu, Amanda C. Tep, Bailey Bergue, Catherine Degnin, Yiyi Chen, Shearwood McClelland, Jerry J. Jaboin

**Affiliations:** ^1^ Department of Radiation Medicine Oregon Health & Science University Portland Oregon USA; ^2^ Biostatistics Shared Resource Oregon Health & Science University Portland Oregon USA; ^3^ Department of Radiation Oncology Indiana University Indianapolis Indiana USA

**Keywords:** anaplastic pleomorphic xanthoastrocytoma, National Cancer Database, overall survival, pediatric, pleomorphic xanthoastrocytoma, radiotherapy

## Abstract

**Background:**

Pleomorphic xanthoastrocytomas (PXAs) account for <1% of primary brain tumors, occurring predominantly in children and young adults. Surgical resection serves as the primary treatment for PXAs, while radiotherapy (RT) and chemotherapy protocols remain poorly defined.

**Aim:**

This study aims to determine current care patterns utilized for pediatric patients (≤ 18 years) diagnosed with PXAs and their effect on overall survival.

**Methods:**

The United States National Cancer Database (NCDB) was queried between 2004 and 2015 for pediatric patients (≤18 years) diagnosed with PXAs.

**Results:**

From the 224 qualifying patients, most patients proceeded with surgery only (78.1%), while 11.6% of patients received both adjuvant RT and chemotherapy. In the 2010‐2015 cohort, patients with subtotal resection were associated with poorer prognosis than those with gross‐total resection (hazard ratio = 17.44, 95% confidence interval = 2.10‐144.90, *p* < .001). RT and chemotherapy recipients were similarly associated with poorer survival than those treated with surgery only, with *p*‐values of <.001 and respective hazard ratios of 3.82 (95% confidence interval = 1.85‐7.90) and 6.68 (95% confidence interval = 3.21‐13.89). The key factors impacting the probability of RT delivery involved WHO grade (*p* < .001) and chemotherapy administration (*p* < .001). However, WHO grade alone did not significantly impact survival (*p*‐value = .088).

**Conclusion:**

Maximally safe resection is the current treatment goal for patients with PXAs. RT and chemotherapy are poorly utilized but had a greater role in managing more aggressive cases of PXAs. Additional research focusing on the impact of adjuvant therapies on tumor progression is needed to better guide treatment decisions.

## 1 INTRODUCTION

Pleomorphic xanthoastrocytomas (PXAs) are rare neuroglial tumors that occur predominantly in children and young adults.^1^, ^2^ They account for <1% of primary brain tumors and are considered World Health Organization (WHO) grade II tumors.^1^, ^3^, ^4^, ^5^ They are associated with favorable prognosis with respective 5‐year and 10‐year overall survival (OS) rates of about 77.8% and 68.5%,^2^, ^3^, ^5^ with an approximate 5‐year progression‐free survival (PFS) rate of 68.3%.^2^, ^5^ However, these tumors may transform into anaplastic PXAs (aPXAs) in 15%‐20% of patients.^3^, ^5^, ^6^

aPXAs are defined as a subset of PXAs in the revised 2016 WHO classification for central nervous system (CNS) tumors. These tumors are considered WHO grade III with their increased mitotic activity of ≥5 per high‐powered fields.^1^, ^2^, ^5^, ^7^ They present with a higher likelihood for necrosis, proliferation, absent pericellular reticulin, and infiltration.^2^, ^7^, ^8^, ^9^

Complete surgical resection presently serves as the primary treatment for PXAs,^3^, ^10^ while the role of adjuvant radiotherapy (RT) and chemotherapy in managing this disease remains undefined. To gain a better understanding of care patterns for pediatric PXAs, the current study aims to evaluate the effect of treatment, particularly RT, on OS using the United States National Cancer Database (NCDB).

## 2 METHODS

The NCDB was established in 1988 by the American College of Surgeons Commission on Cancer (CoC) and the American Cancer Society.^11^ It is a hospital‐based cancer registry that collects information on patients receiving care from a CoC‐accredited institution, which comprises 30% of 5000+ hospitals in the United States. Information reported in the NCDB include deidentified sociodemographic, treatment, tumor, and survival data of oncology patients.

Based on the International Classification of Diseases for Oncology, 3rd edition Histology code of 9424/3, a search through the NCDB brain dataset was conducted for pediatric patients (≤18 years) diagnosed with PXAs in 2004‐2015. The data were exported on February 28, 2020, by authors Y.Y., C.B., and Y.C. These authors verified the data to ensure no problem occurred while data export. Descriptive analysis was conducted summarizing patient demographic and clinical characteristics. To increase transparency concerning the impact of patients with missing values in the study analyses, footnotes referring to the inclusion or exclusion of these patients were provided for each table.

Patients were categorized in two treatment groups: “surgery only” and “surgery and RT.” To assess for differences in patient characteristics between treatment groups, Kruskal–Wallis test was used for continuous variables (i.e., age and tumor size); Cochran‐Armitage test was used for three‐level ordinal variables (i.e., comorbidity score); and Fisher's exact test was used for other categorical variables. Analyses concerning extent of resection (EOR) were based on patients diagnosed between 2010 and 2015 as such information was made available in the database. Our variables were analyzed utilizing log rank testing and Kaplan–Meier modeling. OS was defined as the time in months from diagnosis to death or last follow‐up, whichever occurs first. Univariate and multivariable Cox proportional hazard ratio models were fitted to the data. The final multivariable model was determined based on purposeful selection combined with Akaike's information criterion and Bayesian information criterion. All analyses were performed using R‐version 3.6.2. *p*‐values of <.05 were considered statistically significant. Data consisting of <20 patients were deidentified with asterisks (*) and reported only in percentages.

## 3 RESULTS

For the 224 eligible, pediatric patients, an almost equal occurrence was found between males and females with a median diagnosis age of 14 years (Table 1). Most patients identified as Caucasian (63.8%), followed by Black (16.1%) and Hispanic patients (14.7%). Many resided in a region where ≥14% of adults had no high school degree (60.3%) and earned a median income of <$46, 000 (63.8%). Most lived in a metropolitan area (62.1%) and had private insurance (62.5%). Almost an equal number of patients were observed in 2004‐2009 and 2010‐2015, with many presenting without comorbidities (90.2%). The tumor primarily occurred at the temporal lobe (34.4%) and had a median tumor size of 40 mm (range = 5‐988 mm). The WHO grades were predominantly unspecified (68.8%), but WHO grade IV tumors and PXAs (i.e., WHO grade II), respectively, comprised of 12.9% and 11.6%. A limited number of patients were biopsied (8.0%). Most patients underwent surgery (97.3%). Of the 222 patients with known RT delivery, 18.8% received RT at a median dose of 54.0 Gy (range = 5.4‐83.4 Gy). The preferred RT modality was intensity‐modulated RT (35.7%), followed by photon therapy (31.0%). Chemotherapy was administered to 14.7% of patients.

**TABLE 1:** Overall characteristics of pediatric pleomorphic xanthoastrocytomas (*N* = 224)

Variable	*N* (%)
**Age (years, *N* = 224)**	
Median (range)	14 (1‐18)
**Sex (*N* = 224)**	
Male	115 (51.3%)
Female	109 (48.7%)
**Race (*N* = 224)**	
Caucasian	143 (63.8%)
Black	36 (16.1%)
Hispanic	33 (14.7%)
Other	(5.4%)^a*^
**Median income (*N* = 215)**	
<$46 000	143 (63.8%)
≥$46 000	72 (32.1%)
(Missing)^b^	(4.0%)^a*^
**% without high school degrees (*N* = 215)**	
≥14%	135 (60.3%)
<14%	80 (35.7%)
(Missing)^b^	(4.0%)^a*^
**County population (*N* = 215)**	
Metropolitan, >250 000	139 (62.1%)
Nonmetropolitan	76 (33.9%)
(Missing)^b^	(4.0%)^a*^
**Insurance status (*N* = 215)**	
Private	140 (62.5%)
Government	68 (30.4%)
None	(3.1%)^a*^
(Missing)^b^	(4.0%)^a*^
**Year of diagnosis (*N* = 224)**	
2004–2009	113 (50.4%)
2010–2015	111 (49.6%)
**Comorbidities (*N* = 224)**	
0	202 (90.2%)
1	(6.7%)^a*^
≥2	(3.1%)^a*^
**Tumor location (*N* = 224)**	
Temporal lobe	77 (34.4%)
Frontal lobe	46 (20.5%)
Overlapping lesion of brain	29 (12.9%)
Others	27 (12.1%)
Parietal lobe	25 (11.2%)
Occipital lobe	(8.9%)^a*^
**Tumor size (mm, *N* = 161)**	
Median (range)	40 (5–988)
**WHO Grade (*N* = 224)**	
NOS	154 (68.8%)
I	(2.7%)^a*^
II	26 (11.6%)
III	(4.0%)^a*^
IV	29 (12.9%)
**Biopsy (*N* = 222)**	
None	203 (90.6%)
Biopsy, primary site	(8.0%)^a*^
Surgical procedure with a bypass, no biopsy	(0.4%)^a*^
(Missing)^b^	(0.9%)^a*^
**Surgery (*N* = 224)**	
Yes	218 (97.3%)
No	(2.7%)^a*^
**Extent of Surgery (2010–2015 data, *N* = 103)**	
GTR	72 (64.9%)
STR	31 (27.9%)
(Missing)^b^	(7.2%)^a*^
**RT (*N* = 222)**	
No	180 (80.4%)
Yes	42 (18.8%)
(Missing)^b^	(0.9%)^a*^
**RT Dose (GY, *N* = 37)**	
Median (range)	54 (5.4–83.4)
**RT Modality (*N* = 42)**	
Intensity‐modulated RT	(35.7%)^a*^
Photons	(31.0%)^a*^
External beam, NOS	(19.0%)^a*^
Conformal or 3‐D therapy	(7.1%)^a*^
Stereotactic radiosurgery, NOS	(2.4%)^a*^
Gamma Knife	(2.4%)^a*^
Other, NOS	(2.4%)^a*^
(Missing)^b^	(0.05%)^a*^
**Chemotherapy (*N* = 222)**	
No	189 (85.1%)
Yes	33 (14.7%)
(Missing)^b^	(0.9%)^a*^

Abbreviations: GTR, Gross total resection; NOS, not otherwise specified; RT, radiotherapy; STR, subtotal resection.

^a^ Patient populations with <20 patients were reported with asterisks (*) for de‐identification purposes.

^b^ Patients with missing values were included in analyses.

Patients who underwent surgery and had complete RT information comprised of 216 patients, of which 81.0% had surgery only (Table 2). Based on the Fisher's exact tests, factors significantly associated with RT delivery included: WHO grade (*P* < 0.001) and chemotherapy administration (*p* < .001). Most, if not all, PXAs with WHO grades I, II, and III were typically resected without RT (100%, 96%, and 55.6%, respectively), while more WHO grade IV tumors underwent RT postsurgery (55.6%). Those receiving chemotherapy more likely received surgery with RT (83.9%).

**TABLE 2:** Key variables associated with surgery ± Radiotherapy in pediatric pleomorphic xanthoastrocytomas (*N* = 216)

	Surgery	Surgery + RT	*p*‐value
	*N* (Row %)	*N* (Row %)	
**WHO grade**	*N* = 175 (81.0%)	*N* = 41 (19.0%)	<.001
I	(100%)^a*^	(0.0%)^a*^	
II	24 (96%)	(4%)^a*^	
III	(55.6%)^a*^	(44.4%)^a*^	
IV	(44.4%)^a*^	(55.6%)^a*^	
NOS	128 (85.9%)	21 (14.1%)	
**Chemotherapy**	*N* = 174 (80.9%)	*N* = 41 (19.1%)	<.001
No	169 (91.8%)	(8.2%)^a*^	
Yes	(16.1%)^a*^	26 (83.9%)	
(Missing)^b^	(100%)^a*^	0 (0.0%)	

Abbreviations: NOS, Not otherwise specified; RT, radiotherapy.

^a^ Patient populations with <20 patients were reported with asterisks (*) for de‐identification purposes.

^b^ Patients with missing values were excluded from analyses (*N* = 8).

**FIGURE 1:**
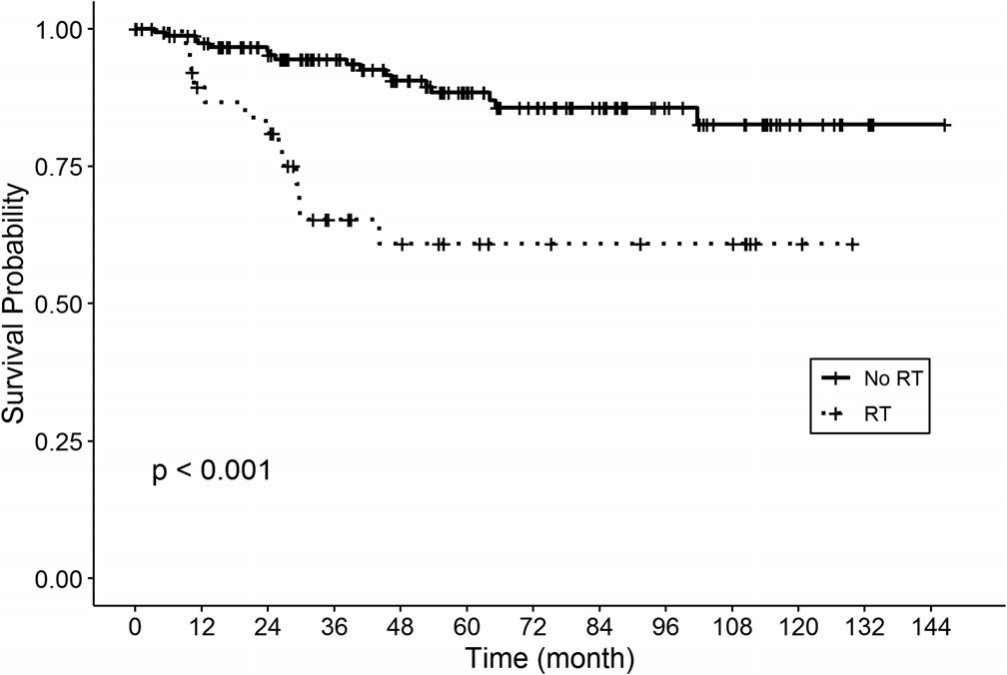
Kaplan–Meier plot of Pediatric Pleomorphic Xanthoastrocytomas

The median OS time of the entire cohort was indeterminate due to the limited number of events (i.e., deaths) observed. However, 3‐ and 5‐year OS rates were calculated for selected groups of subjects (Table 3). The 3‐year OS for patients, respectively, without and with RT were 94.4% (95% confidence interval [CI] = 89.2%‐97.4%) and 65.2% (95% CI = 46.7%‐78.7%). Based on the log rank test and Kaplan–Meier curves, OS is significantly longer for non‐RT compared to RT recipients (*p* < .001, Table 4, Figure 1). Adjuvant chemotherapy administration also affected OS (*p* < .001, Table 4), with higher 3‐year OS for nonchemotherapy (94.8%, 95% CI = 89.9%‐97.4%) versus chemotherapy recipients (51.4%, 95% CI = 30.1%‐69.1%).

**TABLE 3:** Summary of overall survival rates of pediatric pleomorphic xanthoastrocytomas

Variable	*N*	3‐year OS % (95% CI)	5‐year OS % (95% CI)
**Extent of** Surgery^b^, ^c^ (***N* = 82)**			
GTR	60	97.5 (83.5‐99.6)	97.5 (83.5‐99.6)
STR	22	73.7 (47.6‐88.2)	61.5 (29.4‐82.4)
**Extent of Surgery + Chemotherapy** ^b^, ^c^ **(*N* = 82)**			
GTR	56	97.3 (82.3‐99.6)	97.3 (82.3‐99.6)
GTR + Chemotherapy	^a*^	100 (N/A)	N/A
STR	^a*^	85.1 (52.3–96.1)	70.9 (30.9‐90.4)
STR + Chemotherapy	^a*^	40 (5.2‐75.3)	N/A
**Extent of Surgery + RT** ^b^, ^c^ **(*N* = 81)**			
GTR	50	97 (80.4‐99.6)	97 (80.4‐99.6)
GTR + RT	^a*^	100 (N/A)	N/A
STR	^a*^	93.3 (61.3–99.0)	77.8 (31.6‐94.7)
STR + RT	^a*^	33.3 (4.6–67.6)	N/A
**RT (*N* = 199)^c^**			
No	161	94.4 (89.2‐97.2)	88.4 (81.0‐93.1)
Yes	38	65.2 (46.7–78.7)	60.9 (41.7‐75.5)
**Chemotherapy (*N* = 199)^c^**			
No	171	94.8 (89.9–97.4)	89.2 (82.2‐93.5)
Yes	28	51.4 (30.1–69.1)	45 (23.6‐64.2)
**Race (*N* = 201)^c^**			
Caucasian	127	87.5 (79.7‐92.4)	82.5 (73.4‐88.7)
Black	31	96.7 (78.6‐99.5)	92.1 (71.3‐98.0)
Hispanic	32	92.6 (73.5‐98.1)	81.7 (57.1‐93.0)
Other	^a*^	70.1 (32.3‐89.5)	70.1 (32.3–89.5)
**WHO Grade (*N* = 201)^c^**			
I	^a*^	100 (N/A)	100 (N/A)
II	22	95.2 (70.7–99.3)	89.3 (63.2‐97.2)
III	^a*^	75 (31.5–93.1)	60 (19.5‐85.2)
IV	24	70.3 (44.7‐85.7)	70.3 (44.7–85.7)
NOS	143	91.2 (84.7‐95.1)	85.5 (77.1‐91.0)

Abbreviations: CI, Confidence interval; GTR, gross total resection; N/A, not available; NOS, not otherwise specified; OS, overall survival; RT, radiotherapy; STR, subtotal resection.

^a^ Patient populations with <20 patients were reported with asterisks (*) for de‐identification purposes.

^b^ The findings reported are based on the 2010‐2015 data only.

^c^ Patients with missing values were excluded from analyses.

**TABLE 4:** Key variables determined via log rank test for pediatric pleomorphic xanthoastrocytomas

Variables	*N*	*p*‐value
GTR versus STR^a^, ^b^	82	<.001
Extent of Surgery + Chemotherapy^a^, ^b^	81	<.001
Extent of Surgery + RT^a^, ^b^	81	Not reliable
± Adjuvant RT^b^	199	<.001
± Adjuvant Chemotherapy^b^	199	<.001
Race^b^	201	.175
WHO Grade^b^	201	.088

Abbreviations: GTR, Gross total resection; RT, radiotherapy; STR, subtotal resection.

^a^ The findings reported are based on the 2010‐2015 data only.

^b^ Patients with missing values were excluded from analyses.

The 2010‐2015 cohort (*n* = 103) indicated that most had gross‐total resection (GTR, 64.9%) rather than subtotal resection (STR) (Table 1). With only 79.6% of the 2010‐2015 cohort having known survival outcomes, the log rank test revealed GTR impacted survival (*p* < .001, Table 4). Increased OS was observed in patients who underwent GTR rather than STR (3‐year OS = 97.5%, 95% CI = 83.5%‐99.6% vs. 73.7%, 95% CI = 47.6%‐88.2%, Table 3). EOR and chemotherapy also affected survival (*p* < .001, Table 4). Whereas minor differences in the 3‐year OS were observed in patients having GTR with or without chemotherapy, a major difference in 3‐year OS was seen for patients having STR with and without chemotherapy (3‐year OS = 40%, 95%CI = 5.2%‐75.3% vs. 85.1%, 95% CI = 52.3%‐96.1%, respectively). Although there were too few patients to make the log rank test reliable, similar results were observed for those receiving surgery with or without RT (*p* < .001, Table 4). There was little difference in 3‐year OS for patients having GTR with and without adjuvant RT, but increased 3‐year survival was observed in patients undergoing only STR compared to patients proceeding with adjuvant RT (3‐year OS = 93.3%, 95% CI = 61.3%‐99.0% vs. 33.3%, 95% CI = 4.6%‐67.6%).

Univariate cox regression analyses indicated EOR (*p* < .001) and the administration of RT (*p* < .001) and chemotherapy (*p* < .001) independently affect OS (Table 5). STR patients had a higher mortality risk (hazard ratio [HR] = 17.44, 95% CI = 2.10‐144.90) relative to GTR patients. Compared to patients without adjuvant therapies, RT and chemotherapy recipients respectively had HRs of 3.82 (95% CI = 1.85‐7.90) and 6.68 (95% CI = 3.21‐13.89). Only receipt of chemotherapy remained significant in the multivariable model.

**TABLE 5:** Key variables determined via cox regression analyses for pediatric pleomorphic xanthoastrocytomas

Variable	*N*	% of Events	HR (95% CI)	*p*‐value
**Sex** ^**U**^ **(*N* = 201)**				.229
Male	104	10.6%	Reference	
Female	97	19.6%	1.57 (0.75‐3.31)	
**Race** ^**U**^ **(*N* = 201)**				.175
Caucasian	127	15.0%	Reference	
Black	31	6.5%	0.4 (0.09‐1.73)	
Hispanic	32	18.8%	1.46 (0.58‐3.67)	
Other	11	27.3%	2.4 (0.71‐8.14)	
**WHO grade^U^ (*N* = 201)**				.088
I	^a*^	0%	0 (0‐Inf)	
II	22	9.1%	Reference	
III	^a*^	37.5%	4.57 (0.76‐27.40)	
IV	24	25%	3.81 (0.77‐18.96)	
NOS	143	13.3%	1.57 (0.37‐7.75)	
**Surgery** ^**U**^ **(*N* = 201)**				.533
No	^a*^	25%	Reference	
Yes	197	14.7%	0.54 (0.07‐3.95)	
**Extent of surgery** ^b^ ^**,U**^ **(*N* = 82)**				<.001
GTR	60	1.7%	Reference	
STR	22	27.3%	17.44 (2.10–144.90)	
(Missing)^c^	^a*^	16.7%	N/A	
**RT** ^**U**^ **(*N* = 199)**				<.001
No	161	10.6%	Reference	
Yes	38	34.2%	3.82 (1.85–7.90)	
(Missing)^c^	^a*^	0.0%	N/A	
**Chemotherapy** ^**U,M**^ **(*N* = 199)**				<.001
No	171	9.9%	Reference	
Yes	28	46.4%	6.68 (3.21–13.89)	
(Missing)^c^	^a*^	0.0%	N/A	

Abbreviations: CI, Confidence interval; GTR, gross total resection; HR, hazard ratio; RT, radiotherapy; STR, subtotal resection; ^U^, Univariate; ^M^, Multivariable.

^a^ Patient populations with <20 patients were reported with asterisks (*) for de‐identification purposes.

^b^ The findings reported are based on the 2010‐2015 data only.

^c^ Patients with missing values were excluded from analyses.

## 4 DISCUSSION

PXAs were first detailed by Kepes et al in 1979.^4^, ^5^, ^12^ However, due to the infrequent occurrence of this disease, current literature concerning PXAs comprise primarily of case reports and scarce case series.^3^, ^5^ The publication on PXAs by Perkins et al^3^ (*n* = 215) resembles the present study, as it examined the effects of demographic, clinical, and treatment variables on OS for patients with PXAs. Perkins et al queried the Surveillance, Epidemiology, and End Results cancer registry for children and adults diagnosed with PXAs between 1981 and 2007. Relative to Perkins et al, the present study contains twice the number of pediatric patients. The present study's focus on children over the past 12‐years also limited the effect of temporal bias, as it confined diagnostic and treatment variation on a multi‐institutional level.

Even though Perkins et al^3^ included both children and adults in their study, they similarly observed that most patients identified as Caucasian (80%). Their data also indicated that race did not impact survival (*p* = .40). Our analyses further indicated likelihood of RT delivery and mortality risk were not significantly associated with patient demographics. RT delivery was more impacted by WHO grade and chemotherapy delivery, while significant mortality risk was associated with EOR and administration of RT and chemotherapy.

Histologically, PXAs are characterized by astrocytes that usually have large mono‐ or polynucleated structures and spindle cells with a mesenchymal appearance oriented in intersecting bundles.^1^, ^6^ They are distinguishably pleomorphic with some containing lipid droplets. They typically have granular bodies differing in texture, size, and eosinophilia, in addition to the rare observation of some mitotic activity and a general lack of necrosis.^5^, ^6^, ^8^ aPXAs have an increased mitotic activity, which is observed as necrosis and hypercellularity.^6^ Presently, aPXAs are only diagnosed based on histopathologic findings, and the mechanisms that result in its malignancy are unknown.^8^ In the present study, only 8.0% of patients in the overall cohort underwent biopsy, but 4.0% and 12.9% were respectively classified as aPXAs and WHO grade IV tumors. The discrepancy between the number of biopsied patients and reported high‐grade PXAs raises concerns regarding the putative diagnosis of PXAs in the NCDB, as the current standard practice of diagnosing high‐grade PXAs requires histological confirmation.

PXAs are strictly defined as WHO grade II (i.e., PXAs) and WHO grade III (i.e., aPXAs) tumors, according to the 2016 WHO classification for CNS tumors. However, cancer databases include WHO grade I and IV tumors in PXA cohorts. When considering the occurrence of PXA in all WHO grades, Perkins et al^3^ reported the highest frequency of PXAs (19%), followed by aPXAs (12%), WHO grade IV lesions (11%), and WHO grade I tumors (4%). Most PXAs (54%) noted by Perkins et al,^3^ however, had unknown WHO grades. By contrast, the present study contained a higher rate of WHO grade IV tumors (12.9%), followed by PXAs (11.6%), aPXAs (4.0%), and WHO grade I lesions (2.7%). Nonetheless, like Perkins et al, the present study observed most tumors having unknown WHO grades (68.8%). The rationale behind the diagnosis of PXAs as WHO grade I and IV tumors in cancer databases warrant evaluation in order to determine the appropriateness of these tumor grades, as these tumor grades fall outside the present WHO classification system for PXAs.

PXAs may be erroneously diagnosed as glioblastomas (GBMs).^8^, ^13^, ^14^, ^15^ GBMs are commonly mistaken for aPXAs, as they appear similar in radiographic images and exhibit high mitotic activity and frequent necrosis.^16^, ^17^ The absence of appropriate nuclear pleomorphism, abundant reticulin sites, lymphocytic infiltration, and lipid‐filled cytoplasms are also associated with aPXAs and a variety of GBMs.^16^, ^18^ By contrast, patients with PXA WHO grade IV tumors exhibit longer median OS than patients with GBMs (3.75 years vs. 6 months, *p* < .0001).^3^ *BRAF V600E* mutations typically do not occur in GBM cases, whereas one study observed such mutations were more prominent in PXAs and aPXAs in relation to other CNS tumors in adults and children.^7^, ^19^ The presence of such mutations may serve as a useful marker to differentiate PXAs from GBM but warrant further investigation.^19^ Due to the challenges of differentiating between PXAs and GBMs, some PXAs may be misdiagnosed as GBMs and vice versa. This may also explain the consideration of some PXAs as WHO grade IV tumors in the NCDB.

*BRAF V600E* mutations were reported to occur in 65% and 78% of PXAs.^2^, ^8^, ^9^, ^19^ These mutations also appear to occur at an equal rate between PXAs and aPXAs.^9^, ^19^ In a study's PXA cohort (*N* = 87), Schindler et al^19^ observed that these mutations appeared more prevalent in children with aPXAs (100%) than in adults with aPXAs (38%). These mutations arguably may serve as a reasonable diagnostic marker for aPXAs. The presence of *BRAF V600E* mutations was associated with increased OS in relation to *BRAF V600E* nonmutant tumors in general PXA cases (*p* = .02).^2^ However, Schindler et al postulated that such an increased survival may be due to a higher frequency of *BRAF V600E* mutations in PXAs (75.0%,) rather than aPXAs (47.4%). Multivariable analysis based on WHO grade and presence of these mutations were encumbered by the small number of events in the study series.

Tonse et al^20^ found that out of 27 patients, 48% of patients with either PXAs or aPXAs expressed the *BRAF V600E* mutation. Comparable OS was observed between patients with and without *BRAF V600E* mutations, and reduced PFS rates were observed in patients with *BRAF V600E* mutations (3‐year estimates: 51.9% vs. 73%), although this did not achieve statistical significance.

Philips et al and Koelsche *et al* ^8^, ^9^ considered that in addition to *BRAF V600E* mutations, *CDKN2A* deletions are common in PXAs. The former study also indicated that RAF alternations may equally serve as potential genetic marker for PXAs and aPXAs as such alterations were present in all PXA cases in the study cohort.^8^, ^9^ A recent study by Vaubel et al^21^ (*N* = 64) reported a higher occurrence of *CDKN2A/*B deletions (94%) relative to *BRAF V6000E* (76.1%). The inclusion of the presence of these mutations in patient datasets in the NCDB will enhance future analyses concerning PXAs and improve the current understanding of how these mutations affect patient outcomes, such as OS.

Mallick et al^22^ observed respective median OS of 209 and 49 months for PXAs and aPXAs (*N* = 325). Univariate cox regression analyses indicated that relative to PXAs, aPXAs had increased risk of progression (HR = 3.18, 95% CI = 1.6‐6.4, *p* = .001) and death (HR = 2.2, 95% CI = 1.4‐3.6, *p* = .001). Multivariable analyses confirmed this increased risk of progression in aPXAs compared to PXAs (HR = 2.0, 95% CI = 1.2‐3.3, *p* = .005). Tonse et al^20^ similarly observed an increased risk of progression with aPXAs (*N* = 37, HR = 4.5, 95% CI = 1.37‐14.83, *p* = .013) in univariate analyses. Ida et al^2^ found that aPXAs had lower 5‐year OS than PXAs (*N* = 74, 57.1% vs. 90.4%, *p* = .0003). Vaubel et al^21^ reported higher 5‐year OS in PXAs relative to aPXAs (80.8% vs. 47.6%, *p* = .0009). Similarly, univariate cox analysis from the current study indicated increased mortality risk in aPXAs relative to PXAs (HR = 4.57, 95% CI = 0.76‐27.4, *p* = .088). We also observed longer 3‐year OS for PXAs than aPXAs (3‐year OS = 95.2%, 95% CI = 70.7%‐99.3% vs. 75%, 95% CI = 31.5%‐93.1%).

Similar to the pediatric cohort of Ida et al^2^ (*N* = 31), the present study indicated surgical resection remains the mainstay therapy for pediatric patients with PXAs (97.3%). Based on a univariate analysis from the publication of Perkins et al,^3^ patients without surgical resection exhibited increased mortality risk than those with surgery (HR = 2.80, 95% CI = 0.99‐7.97, *p* = .05). The present study did not observe a significant difference in survival between patients with and without surgical resection (*p* = .533). This finding may have been affected by the low number of patients that did not undergo resection.

Several studies have noted that EOR affects patient outcomes. Perkins et al^3^ observed that 68% of PXA patients had GTR, while 22% had either debulking or partial resection. The latter group were not significantly associated with survival outcomes in univariate (HR = 1.48, 95% CI = 0.82‐2.7, *p* = .19) or multivariable analyses (HR = 1.26, 95% CI = 0.69‐2.30, *p* = .45). Similar HRs were seen by Mallick et al.^3^, ^22^ However, Mallick et al^22^ noted significant differences in OS between patients with GTR and STR in univariate and multivariable cox regression analyses with respective *p*‐values of .017 and .010. Mallick et al reported 56.1% and 31.4%, respectively, underwent GTR and STR. Compared to patients that proceeded with GTR, patients undergoing STR had increased risk for progression in univariate analyses (HR = 2.19, 95% CI = 1.1‐4.2, *p* = .019) and multivariable analyses (HR = 1.9, 95% CI = 1.1‐3.2, *p* = .018).

Although the NCDB only provided information on EOR from 2010 to 2015, the current study observed that 69.9% of patients with known EOR underwent GTR, while the remaining 30.1% underwent STR. Like prior studies, EOR was shown to impact survival (*p* < .001, log‐rank test; *p* < .001, univariate cox regression). Patients with STR were associated with decreased survival compared with GTR (HR = 17.44, 95% CI = 2.10‐144.90). Similarly, reduced 3‐year OS was observed in patients proceeding with STR versus GTR (73.7%, 95% CI = 47.6‐88.2% vs. 97.5%, 95% CI = 83.5%‐99.6%).

Perkins et al^3^ noted RT was administered in 25% of the study cohort. Increased mortality risk was observed in RT versus non‐RT recipients in univariate (HR = 4.47, 95% CI = 2.61‐7.66, *p* < .0001) and multivariable analyses (HR = 3.66, 95% CI = 2.06‐6.41, *p* < .0001).^3^ In this study, multivariable analyses were adjusted for statistically significant variables in the univariate analyses with *p* < .10 level (e.g., surgery, RT, age and sex). The study suspected the increased risk in patients undergoing RT may be attributed to most RT patients having high‐grade tumors (51%). Ida et al^2^ found that 47.3% of patients within their study received some form of postoperative therapy. They similarly observed that adjuvant therapies were more common in patients with aggressive tumors, which they classified based on patients experiencing an early event (i.e., recurrence or death within 3 years from initial diagnosis). RT administration was provided to 57.1% of patients with early events as opposed to 17.1% without early events (*P* = 0.003). Chemotherapy was delivered in 61.9% of patients with early events as opposed to 22.39% without (*p* = .005). Most patients who experienced an early event typically underwent STR/Biopsy, had tumors with a mitotic index of ≥5/10 HPF, and were diagnosed with aPXAs (*p* = .004, *p* = .02, and *p* = .008, respectively).

In the present study, RT and chemotherapy were administered in 18.8% and 14.7% of patients, respectively. Like Perkins et al,^3^ RT recipients exhibited increased mortality risk over those without RT in our univariate analysis (HR = 3.82, 95% CI = 1.85‐7.9, *p* < .001). Patients who received chemotherapy had increased mortality risk compared to those who did not receive chemotherapy (*p*‐value < .001, log‐rank test; HR = 6.68; 95% CI = 3.21‐13.89, *p*‐value<.001, univariate cox regression). Chemotherapy was commonly administered to RT recipients (63.4%, *p* < .001). WHO grade was frequently associated with RT administration (*p* < .001). WHO grade IV tumors likely received postoperative RT, while PXAs primarily underwent surgery only. These findings suggest that poor patient outcomes may be more attributed to the aggressive nature of high‐grade tumors rather than the administration of RT and chemotherapy, considering that such therapies were mostly utilized for WHO grade IV tumors in the study cohort.

Selection bias continues to be an issue in this study, since the cases in the NCDB are comprised of patients diagnosed and treated in facilities accredited by the Commission on Cancer.^23^ Additionally, although the NCDB comprises 72% of newly diagnosed cancer cases, the rarity of PXAs, the prevalence of missing information (e.g., EOR prior to 2009, WHO grade, and tumor size), and the poor utilization of adjuvant therapies limit the sample size and statistical power of the analyses. The NCDB reports on patients diagnosed outside of the WHO classification of PXAs, drawing concerns toward the misdiagnosis of WHO grade I and IV PXA cases. The potential for misdiagnosis is further increased by the limited information on the central review of pathology and molecular data in the NCDB. Vital statuses of patients diagnosed in 2015 could not be included in the 3‐ and 5‐year survival analyses, censoring findings early in the data collection stage. Furthermore, since the NCDB contains information on newly diagnosed cancer cases, the impact of demographic, clinical, and treatment factors on PFS could not be evaluated.^11^ However, PFS remains a valuable outcome to evaluate in a condition that has a high tendency to recur.^4^, ^20^, ^23^

Presently, maximally safe resection is the primary treatment recommendation for PXAs.^3^, ^20^, ^22^ Observation for younger patients who undergo GTR is a reasonable approach, as EOR and young age are considered significant factors in improved PFS and OS.^20^, ^22^ Surveillance MRIs of the brain with contrast‐enhancement is recommended at 3‐month intervals during the first 3 years from diagnosis, biannually the following 2 years, and annually thereafter.^22^ If the tumor recurs, patients should pursue aggressive treatment, such as secondary surgery and RT.^20^ For tumors with atypical features, administrating postoperative therapy, such as RT and chemotherapy, may be beneficial considering these tumors are associated with reduced survival and a higher likelihood for recurrence than PXAs.^3^, ^22^

## 5 CONCLUSION

Maximally safe resection is the mainstay treatment for patients with PXAs. The role of adjuvant therapies, such as RT and chemotherapy, remains poorly defined but is greatly utilized post surgery for patients with high‐risk disease. Further investigation is warranted to determine the use of adjuvant therapies in managing and minimizing tumor progression in PXAs.

## CONFLICT OF INTEREST

The authors have stated explicitly that there are no conflicts of interest in connection with this article.

## AUTHOR CONTRIBUTIONS

All authors had full access to the data in the study and take responsibility for the integrity of the data and the accuracy of the data analysis. *Conceptualization*: Jerry J. Jaboin. *Methodology*: Jerry J. Jaboin, Daphne B. Scarpelli, Yun Yu, Catherine Degnin, and Yiyi Chen. *Investigation*: Daphne B. Scarpelli, and Yun Yu. *Formal Analysis*: Daphne B. Scarpelli, Yun Yu, Catherine Degnin, and Yiyi Chen. *Resources*: Jerry J. Jaboin, Shearwood McClelland III, Yun Yu, Catherine Degnin, and Yiyi Chen. *Writing—Original Draft*: Jerry J. Jaboin, Daphne B. Scarpelli, Amanda C. Tep, and Bailey Bergue. *Writing—Review and Editing*: Jerry J. Jaboin, Shearwood McClelland III, Daphne B. Scarpelli, Amanda C. Tep, Bailey Bergue, Yun Yu, Catherine Degnin, and Yiyi Chen. *Visualization*: Daphne B. Scarpelli, Amanda C. Tep, Bailey Bergue, Yun Yu, Catherine Degnin, and Yiyi Chen. *Supervision*: Jerry J. Jaboin, Shearwood McClelland III. *Funding Acquisition*: Jerry J. Jaboin.

## ETHICS STATEMENT

The authors state that they have followed the principles outlined in the Declaration of Helsinki for all human experimental investigations. The Oregon Health and Science University institutional review board approved this study.

## Data Availability

The data that support the findings of this study are available from the corresponding author upon reasonable request.
